# Impact of Erector Spinae Muscle Mass on Recovery From Tube Feeding in Older Patients With Dysphagia

**DOI:** 10.1111/ggi.70487

**Published:** 2026-04-10

**Authors:** Midori Miyagi, Hideki Sekiya, Satoru Ebihara

**Affiliations:** ^1^ Department of Rehabilitation Medicine Tohoku University Graduate School of Medicine Sendai Japan; ^2^ Nutrition Therapy Center, Toho University Omori Medical Center Tokyo Japan

Dear Editor,

1

Dysphagia arises from a complex interplay of factors and patient recovery from the condition is also multi‐factorial. Previous studies have identified younger age, lower baseline dysphagia severity, higher body mass index (BMI) [1], and stronger tongue pressure [2] as factors associated with recovery to complete oral intake (COI; defined as a Functional Oral Intake Scale (FOIS) score of ≥ 4). In our previous research involving patients aged ≥ 65 years, we demonstrated that the pectoralis muscle index (PMI) derived from chest CT images determines FOIS scores and that osteoporosis is associated with dysphagia severity [3]. Additionally, the erector spinae muscle index (ESMI) reflects skeletal muscle mass index (SMI) and sarcopenia, serving as a prognostic factor in respiratory diseases [4, 5]. Therefore, we hypothesized that these parameters might also predict recovery in patients with severe dysphagia. We tested the hypothesis that skeletal muscle‐related factors serve as predictive factors for recovery to COI at the endpoint of the dysphagia rehabilitation team intervention in patients with severe dysphagia (FOIS levels 1 and 2). In this study, we divided patients with severe dysphagia into a COI group (patients who achieved COI at the endpoint) and a no‐COI group (patients who did not achieve COI at the endpoint) based on the FOIS score at the endpoint of the dysphagia rehabilitation team intervention and analyzed the differences.

We retrospectively reviewed the charts of 211 patients aged ≥ 65 years referred to the dysphagia rehabilitation team at Toho University Omori Medical Center between April 2019 and March 2022. We excluded patients with severe dementia (Mini‐Mental State Examination score < 10) [6], neurodegenerative disease, apparent cerebral infarction, previous head and neck cancer surgery, or missing thoracic CT data. Ultimately, 90 patients with severe dysphagia were chosen for further analysis. Dysphagia severity was assessed using FOIS, integrating findings from videofluoroscopy and videoendoscopy. We measured the cross‐sectional area of the erector spinae muscle (ESM_CSA_) at the Th12 level using a single CT slice. The ESM_CSA_ was characterized using a pixel attenuation of −29 to +150 HU, and ESMI was calculated as ESM_CSA_ divided by height squared.

Of the 90 patients, 52 (57.8%) recovered to COI. The ESMI of the COI group was significantly higher than that of the no‐COI group (Table S1). Endpoint FOIS scores were positively correlated with ESMI (*p* < 0.002, *r* = 0.32) (Figure 1). Multivariate logistic analyses, adjusted for age, sex, BMI, and baseline FOIS, revealed that ESMI (odds ratio 1.307, 95% CI 1.067–1.600; *p* = 0.01) remained an independent predictive factor for achieving COI.

**FIGURE 1:**
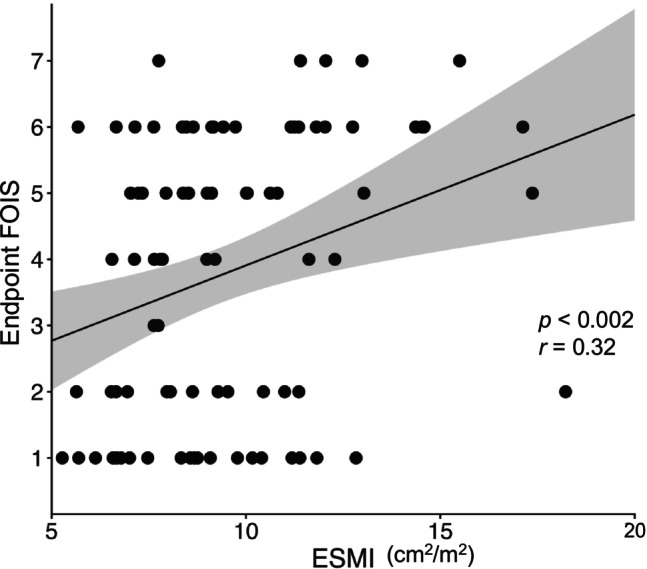
Scatter plot showing the association between the endpoint of FOIS and ESMI. Endpoint FOIS scores were positively correlated with ESMI (*p* < 0.002, *r* = 0.32). ESMI: the erector spinae muscle index; FOIS: the functional oral intake scale.

Based on the analyses above, we conclude that ESMI is an independent predictive factor for recovery to COI in older patients with severe dysphagia. While our previous research highlighted PMI and osteoporosis [3], this study demonstrates that trunk skeletal muscle mass, assessed via ESMI, is crucial for functional recovery. Unlike previous studies that focused on nutritional status [1], we evaluated the posterior trunk muscles, which function as anti‐gravity supports. The independent association of ESMI with recovery suggests it may be a more sensitive predictor than the mass of the pectoralis muscle [7]. The present study was a single‐center study. Future research needs to analyze patients with dysphagia from multiple centers and clarify the mechanism of how ESMI at admission contributes to the subsequent course of dysphagia. In light of ESMI having been shown to be associated with sarcopenia, our results suggest pre‐admission sarcopenia acts as a barrier to achieving COI, potentially encompassing elements of sarcopenic dysphagia. Therefore, comprehensive interventions based on admission chest CT, incorporating both dysphagia rehabilitation and sarcopenia management, should be developed and implemented.

## Author Contributions

Conceptualization: S.E. and M.M. Methodology: S.E. and M.M. Data acquisition: M.M. and H.S. Writing – original draft preparation: M.M. Writing – review: H.S. and S.E. Drafting the work or revising it critically for important intellectual content; final approval of the version to be published; agreement to be accountable for all aspects of the work: All authors.

## Funding

This study was supported by 30th Umami Research Grant from the Society for Research on Umami Taste, Japan; The 3rd JGS Grant for Geriatric Nutrition Research; Japan Society for the Promotion of Science (JSPS) KAKENHI [24K20428] grants awarded to M.M., JSPS KAKENHI (grants 22K19760, 24K02778, and 25K22899 awarded to S.E); the Japan Agency for Medical Research and Development (25zf0127001h0005 to S.E.); the Japan Science and Technology Agency (JST) Strategic International Collaborative Research Program (SICORP) (JPMJSC2308 to S.E.); a Research Funding for Longevity Sciences grant from the National Center for Geriatrics and Gerontology (25‐29 to S.E.), and a grant from the Japan Association of Geriatric Health Service Facilities (Dysphagia Statement Project to S.E.).

## Ethics Statement

The Ethics Committee at Toho University Omori Medical Center approved the study protocol (No. M22112) and informed consent was obtained from all participants in the study using an opt‐out process.

## Consent

Patient consent was obtained using an opt‐out procedure in accordance with the protocol approved by the institutional ethics committee.

## Conflicts of Interest

The authors declare no conflicts of interest.

## Supporting information


**Table S1:** Comparison of clinical characteristics between patients who achieved and not achieve COI.

## Data Availability

The data that support the findings of this study are available from the corresponding author upon reasonable request.
