# Supplementary material for: Impact of Erector Spinae Muscle Mass on Recovery From Tube Feeding in Older Patients With Dysphagia
Source: Geriatr Gerontol Int. 2026 Apr 10;26(4):e70487. doi: 10.1111/ggi.70487 (PMC13068630; doi:10.1111/ggi.70487)
Supplement: Supplementary file 1 — Table S1: Comparison of clinical characteristics between patients who achieved and not achieve COI. [file GGI-26-0-s001.docx]

**Supplementary Table**

**Table S1. Comparison of clinical characteristics between patients who achieved and not achieve COI**

|  | No-COI  n = 38 | COI  n = 52 | p |
| --- | --- | --- | --- |
| Age (y) | 79.7 ± 7.1 | 80.8 ± 7.1 | ns^a^ |
| Female/Male (n) | 13/25 | 25/27 | ns^b^ |
| BMI (kg/m^2^) | 20.2 ± 5.1 | 20.6 ± 4.1 | ns^a^ |
| FOIS score: 1 (%) | 55.2 | 40.4 | ns^a^ |
| Etiology (n)  Pulmonary disease  Cardiac disease  Cancer (without lung)  Lung cancer  Dermatosis  Digestive system disease  Renal urological disease  Endocrine disease  Meningitis  Orthopedic disease  Psychiatric disease  Others | 15  9  4  1  3  2  1  0  0  0  0  3 | 15  11  4  3  0  3  5  5  1  1  1  3 | ns^b^  ns^b^  ns^b^  ns^b^  ns^b^  ns^b^  ns^b^  ns^b^  ns^b^  ns^b^  ns^b^  ns^b^ |
| Comorbidities (n)  　Hypertension  　Diabetes mellitus  Cerebral vascular disease  Chronic kidney disease  Cognitive disorder  Heart failure  　Cancer  COPD  Collagen disease  Others | 16  10  8  3  7  3  1  2  1  5 | 16  18  9  4  4  4  6  4  2  2 | ns^b^  ns^b^  ns^b^  ns^b^  ns^b^  ns^b^  ns^b^  ns^b^  ns^b^  ns^b^ |
| Image measurements  PM_CSA_ (cm^2^)  PMI (cm^2^/m^2^)  ESM_CSA_ (cm^2^)  ESMI (cm^2^/m^2^) | 23.3 ± 9.4  9.0 ± 3.4  22.1 ± 7.6  8.5 ± 2.7 | 23.6 ± 7.3  9.6 ± 3.1  24.2 ± 7.5  9.9 ± 3.0 | ns^a^  ns^a^  ns^a^  0.018 ^a^ |

Values are shown as means ± standard deviations or numbers. ^a^ Mann–Whitney U test; ^b^ χ^2^ test. COI, complete oral intake; BMI, body mass index; PM_CSA_, cross-sectional area of pectoralis muscle; PMI, pectoralis muscle index; ESM_CSA,_ cross-sectional area of erector spinae muscle; ESMI, erector spinae muscle index; ns, not significant.
